# Diet composition, not calorie intake, rapidly alters intrinsic excitability of hypothalamic AgRP/NPY neurons in mice

**DOI:** 10.1038/srep16810

**Published:** 2015-11-23

**Authors:** Wei Wei, Kevin Pham, Jesse W. Gammons, Daniel Sutherland, Yanyun Liu, Alana Smith, Catherine C. Kaczorowski, Kristen M.S. O’Connell

**Affiliations:** 1 Department of Physiology, University of Tennessee Health Science Center, Memphis, TN 38163; 2 College of Basic Medicine, Hubei University of Chinese Medicine, Wuhan City, Hubei Province, China 430065; 3 Department of Anatomy and Neurobiology, University of Tennessee Health Science Center, Memphis, TN 38163; 4 The Neuroscience Institute, University of Tennessee Health Science Center, Memphis, TN 38163.

## Abstract

Obesity is a chronic condition resulting from a long-term pattern of poor diet and lifestyle. Long-term consumption of high-fat diet (HFD) leads to persistent activation and leptin resistance in AgRP neurons in the arcuate nucleus of the hypothalamus (ARH). Here, for the first time, we demonstrate acute effects of HFD on AgRP neuronal excitability and highlight a critical role for diet composition. In parallel with our earlier finding in obese, long-term HFD mice, we found that even brief HFD feeding results in persistent activation of ARH AgRP neurons. However, unlike long-term HFD-fed mice, AgRP neurons from short-term HFD-fed mice were still leptin-sensitive, indicating that the development of leptin-insensitivity is not a prerequisite for the increased firing rate of AgRP neurons. To distinguish between diet composition, caloric intake, and body weight, we compared acute and long-term effects of HFD and CD in pair-fed mice on AgRP neuronal spiking. HFD consumption in pair-fed mice resulted in a significant increase in AgRP neuronal spiking despite controls for weight gain and caloric intake. Taken together, our results suggest that diet composition may be more important than either calorie intake or body weight for electrically remodeling arcuate AgRP/NPY neurons.

In humans, most obesity is caused by an imbalance between food intake and energy expenditure. Ordinarily, mammals, including humans, are quite efficient at matching caloric intake to energy expenditure, particularly over the long-term^1^,^2^,^3^,^4^. Body weight and adiposity are thought to be maintained at a “setpoint”—likely determined by the activity of neurons within the hypothalamus. However, this setpoint is not fixed and can be altered in response to exposure to an obesogenic environment (e.g., energy-dense foods or sedentary lifestyle), resulting not only in obesity, but also difficulty losing weight and keeping it off, as the brain now regards the higher body weight as the new “setpoint” and endeavors to defend it. Consistent with this hypothesis, many people ultimately regain lost weight following a successful weight loss program^5^. From this perspective, common obesity can be thought of as a disorder of energy expenditure in which the hypothalamic body weight setpoint is abnormally high and permits intake to substantially exceed expenditure over the long-term.

In the brain, the arcuate nucleus of the hypothalamus (ARH) is essential for the regulation of food intake and energy homeostasis. In the ARH, a well-described microcircuit consisting of anorexigenic neurons expressing pro-opiomelanocortin (POMC) and orexigenic neurons that coexpress agouti-related peptide and neuropeptide Y (AgRP/NPY) is critical for energy balance. Activation of AgRP/NPY neurons is associated with increased food intake and positive energy balance^6^,^7^,^8^,^9^,^10^,^11^ while POMC neuronal activation is associated with satiety and increased energy expenditure^7^,^10^,^12^,^13^,^14^. Both AgRP/NPY and POMC neurons are modulated by a variety of peripheral factors, perhaps the best characterized of which is leptin, which potently inhibits AgRP/NPY neurons^12^,^15^,^16^ and activates POMC neurons^12^. Leptin is important for long-term regulation of body weight and energy homeostasis^17^, but there is mounting evidence that the development of leptin insensitivity in the hypothalamus is a significant contributor to obesity^18^,^19^,^20^,^21^.

We recently showed that diet-induced obesity (DIO) significantly increases the intrinsic excitability of ARH AgRP/NPY neurons, resulting in persistently increased activity that is refractory to inhibition by leptin^22^. In this study, our goal was to investigate the onset of the neuronal changes associated with DIO and determine whether the defects in the AgRP neuronal microcircuit arise as a result of increased body weight, high-fat diet (HFD), or both. We show here that changes in both AgRP neuronal excitability and projections to the paraventricular nucleus (PVH) occur rapidly upon brief exposure to an HFD and provide evidence that diet composition may play a more significant role in remodeling the electrical properties of AgRP/NPY neurons than body weight or caloric intake. Further, we show that the development of leptin-resistance does not contribute significantly to these early diet-related changes, as AgRP/NPY neurons from mice briefly fed HFD are still robustly inhibited by leptin, suggesting that the diet itself influences the function of AgRP/NPY neurons.

## Results

### Short-term consumption of a high-fat diet induces persistently elevated output in arcuate AgRP/NPY neurons

In rodents, AgRP/NPY neuronal electrical activity (e.g., action potential frequency) is exquisitely sensitive to nutritional status: AgRP/NPY neurons from satiated mice fire quite slowly (<1 s^−1^), while those from food-deprived (hungry) mice fire significantly faster (>3 s^−1^)^22^. This switch in AgRP/NPY neuronal excitability is likely crucial for physiological regulation of appetitive behaviors, as direct optogenetic^9^,^10^,^23^ or chemogenetic^11^,^24^,^25^,^26^ manipulation of these neurons has a rapid, potent impact on food intake in mice. We recently demonstrated that long-term (>8 weeks) consumption of a high-fat diet (HFD) dramatically remodels the intrinsic excitability of AgRP neurons—in brain slices from mice fed HFD for at least 8 weeks, AgRP/NPY firing was persistently increased, regardless of nutritional status, and was resistant to inhibition by leptin^22^. Long-term consumption of HFD is associated with other physiological and pathophysiological changes such as obesity, increased adiposity, and altered hormone and nutrient levels^19^,^27^,^28^,^29^, which may influence AgRP/NPY neuronal plasticity and/or leptin-sensitivity. Therefore, in this study, we sought to determine whether these aforementioned changes in AgRP/NPY neuronal output were secondary to other HFD-induced changes (e.g., increased body weight and/or adiposity, hormone levels, etc) or if the persistent activation we observed in our previous study is an early consequence of HFD consumption that precedes and potentially contributes to other sequelae of overweight and obesity. To address this question, we examined the correlation of the diet-induced increase in AgRP/NPY neuronal output with diet-dependent weight gain to ascertain whether weight gain or other factors are required for electrical remodeling of AgRP/NPY neurons and the development of leptin-resistance.

**Figure 1:**
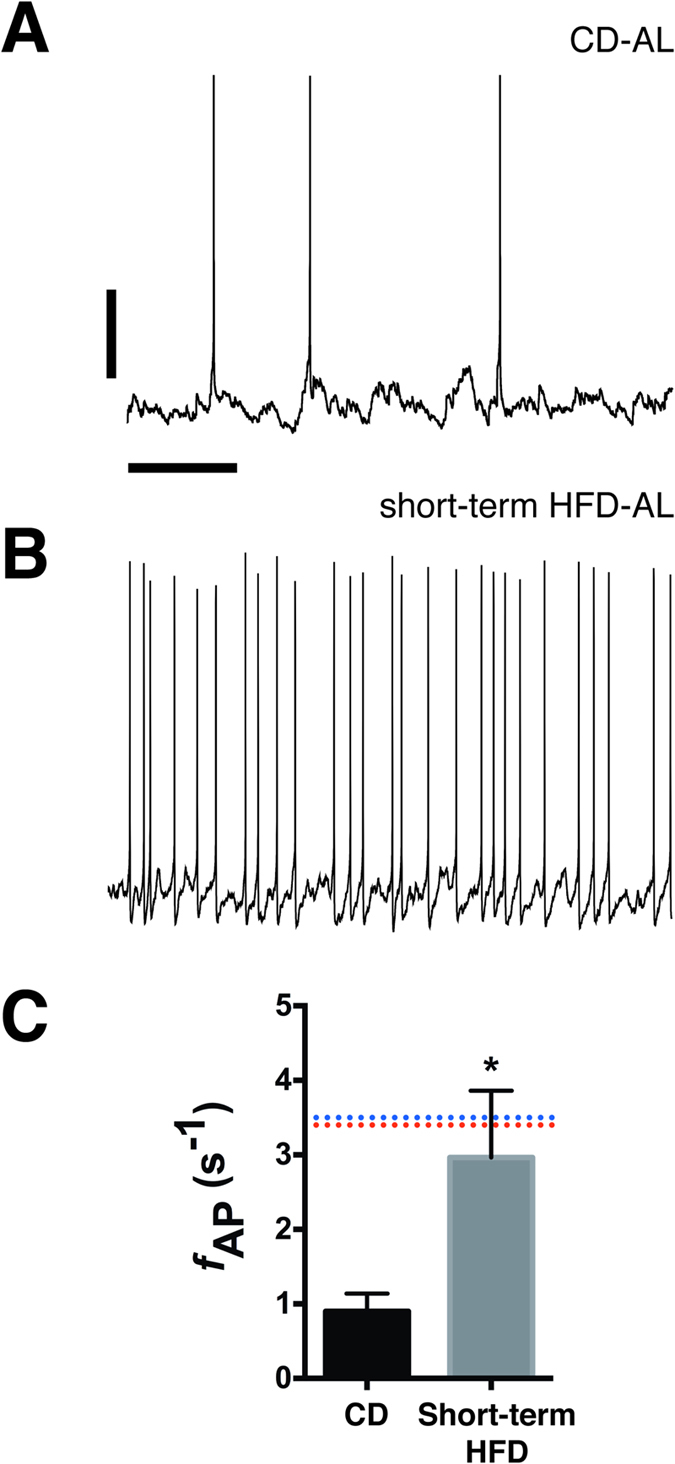
Brief exposure to a high-fat diet induces persistent activation of AgRP neurons in the arcuate nucleus. Representative spontaneous action potentials from AgRP neurons from a CD-fed mouse (**A**) and a mouse fed high-fat diet for 48 h (**B**). Current-clamp recordings were made in the presence of fast synaptic blockers, therefore the recorded action potentials represent the intrinsic electrical activity of these neurons. Scale bars: 20 mV and 2 s. (**C**) Mean action potential frequency from CD and short-term HFD mice. The dashed lines represent the mean firing frequency of AgRP neurons from lean, fasted mice (red) and long-term HFD-fed mice (blue) and are presented for comparison (data from Baver *et al.*, 2014). *p = 0.02, Mann-Whitney U Test.

To better define the timeframe during which feeding mice an HFD promotes inappropriate activation of AgRP/NPY neurons, we fed mice HFD *ad libitum* for 2–6 days and examined the excitability of AgRP/NPY neurons at each timepoint. Consistent with previous reports, we did not observe any significant change in body weight following brief (<6 days) exposure to HFD (see refs 30,31). As shown in Fig. 1, even this short period of HFD feeding was associated with a significant increase in the AP firing rate of AgRP/NPY neurons (CD: 0.9 ± 0.2 s^−1^, n = 7; short-term HFD: 3.0 ± 0.9 s^−1^, n = 16; p = 0.02), similar to what we observed in AgRP/NPY neurons from mice fed HFD long-term, suggesting that HFD-induced remodeling of AgRP/NPY neuronal excitability occurs rapidly following the switch from a lower-fat CD and may be independent of an increase in body weight.

### AgRP/NPY neurons from mice fed HFD short-term are still sensitive to inhibition by leptin

**Figure 2:**
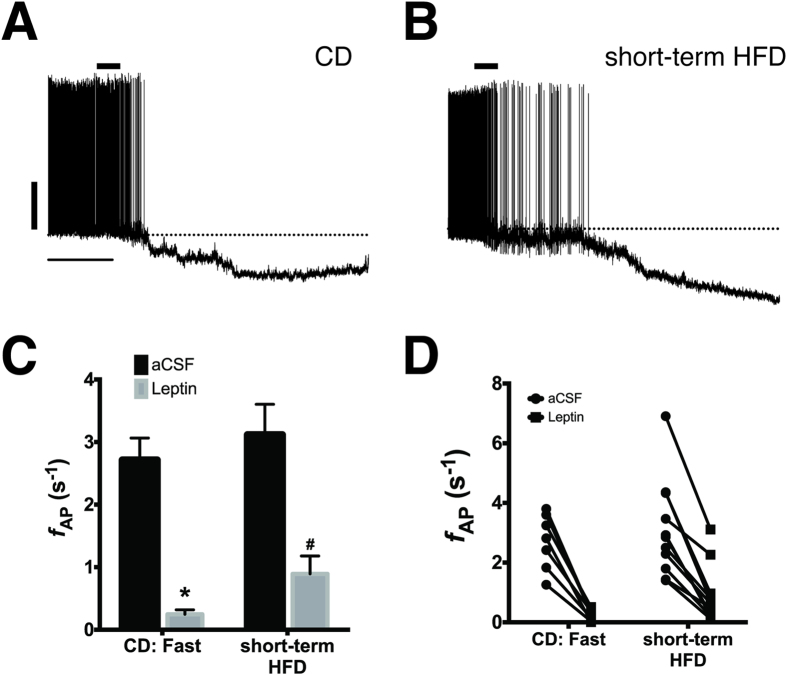
Leptin-dependent inhibition of AgRP neurons is still intact following short-term consumption of HFD. Representative spontaneous action potentials from AgRP neurons from a CD mouse fasted overnight (**A**) and a mouse fed high-fat diet for 4 days (**B**). Leptin (100 nM) was applied for ~1 min where indicated followed by perfusion with normal aCSF. Scale bars: 20 mV and 2 min. In all neurons, regardless of diet, leptin significantly inhibited action potential firing and hyperpolarized the resting membrane potential. (**C**) Mean action potential frequencies before (black) and after (gray) bath perfusion of 100 nM leptin. Group: p < 0.0001, *p = 0.003, ^#^p = 0.01, Kruskal-Wallis Test with Dunn’s *post hoc* multiple comparisons test. (**D**) Plot showing all datapoints from (**C**) before (circles) and after (squares) leptin showing that all neurons in both groups were inhibited by leptin.

In lean mice on a low-fat CD, leptin potently inhibits AP firing in AgRP/NPY neurons^16^,^22^,^32^,^33^,^34^ and this inhibition is significantly blunted in AgRP/NPY neurons from animals fed an HFD long-term^22^. Previous reports suggest that HFD disrupts leptin receptor (LepR) signaling as early as 48 h after the switch from control diet, as assessed by leptin-dependent JAK2 phosphorylation of STAT3 and upregulation of SOCS3^27^,^30^,^31^. Since one possible explanation for the elevated firing rate observed in HFD-fed mice is that leptin fails to inhibit AgRP neuronal firing due to the development of leptin resistance, we determined whether leptin inhibition of electrical activity in AgRP/NPY neurons is also disrupted after short-term HFD consumption. As shown in Fig. 2, bath application of 100 nM leptin to brain slices from CD: fasted mice significantly inhibited AP firing (aCSF: 2.7 ± 0.4 s^−1^; +leptin: 0.24 ± 0.07 s^−1^, p = 0.0001, n = 7), consistent with previous reports^16^,^22^,^34^. Unlike our previous finding in mice fed HFD for at least 8 weeks^22^, 100 nM leptin significantly inhibited AP firing in AgRP/NPY neurons from short-term HFD-fed mice (aCSF: 3.1 ± 0.5; +leptin: 0.9 ± 0.3, p < 0.0001, n = 11; Fig. 2), suggesting that the increased activity of AgRP/NPY neurons in these mice is not a consequence of leptin-resistance.

### Consumption of a high-fat diet reduces AgRP + neuronal projections to the paraventricular hypothalamus

**Figure 3:**
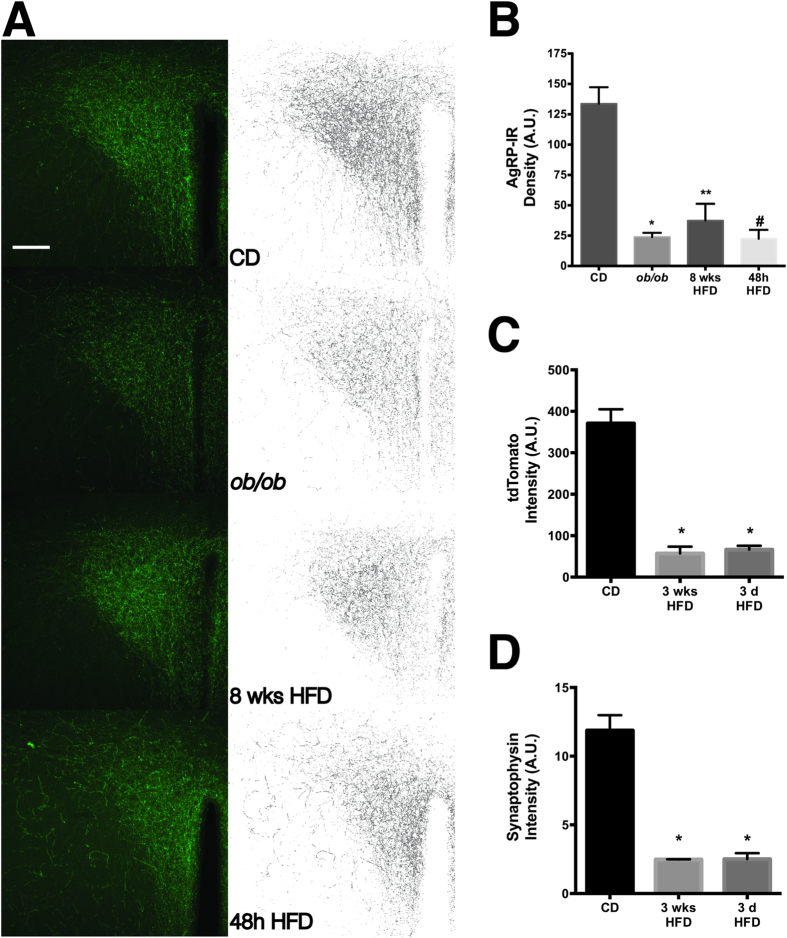
Consumption of high-fat diet in adult mice significantly decreases AgRP-positive neuronal projections to the paraventricular hypothalamic nucleus. (**A**) Representative images of AgRP immunoreactive axonal projections in the PVH from CD, *ob/ob*, 8 weeks HFD, and 48 h HFD mice. The left column is a maximum intensity projection of a 3D image stack and the right column is the skeletonized version of the image. Scale bar = 100 μm. Mean fluorescence intensity for AgRP-IR (**B**), tdTomato (**C**) and Synaptophysin-IR (**D**) for each group calculated from the skeletonized images. Group p = 0.0002, adjusted p-values compared to CD: *p = 0.0004, **p = 0.0009, ^#^p = 0.0003, ANOVA with Tukey’s multiple comparisons *post hoc* test.

Neurons in the PVH are a primary target of AgRP/NPY neurons and inhibition of PVH output by AgRP/NPY neurons is necessary and sufficient for increased feeding in mice^23^. Prior studies of the anatomical projections from ARH → PVH assessed the integrity of this critical pathway in either the offspring of DIO or DIO-resistant rats or in leptin-deficient *ob/ob* mice^35^,^36^. While ARH_AgRP_ → PVH projections are significantly decreased in both of these models, the developmental impact of impaired leptin signaling in both of these models confounds the interpretation of the specific effect of diet and/or body weight on ARH_AgRP_ innervation of the PVH. Therefore, to determine if consumption of a high-fat diet by adult wild-type mice also perturbs innervation of the PVH by ARH AgRP neurons and accompanies the effect we observed on AgRP/NPY neuronal output, we fed mice HFD for either 2 days or 8 weeks, and used AgRP immunoreactivity (AgRP-IR) in the PVH as an indirect measure of ARH to PVH innervation. As expected, we found dense AgRP-IR throughout the PVH in sections from lean, CD-fed mice (Fig. 3A). As previously described^36^, we also observed a significant decrease in AgRP-IR in the PVH from CD-fed *ob/ob* mice (Fig. 3), in support of a role for leptin signaling in AgRP neuronal targeting to the PVH (CD: 133.7 ± 13.6; *ob/ob*: 23.87 ± 3.5, n = 3/group, F(3,8) = 25.8, Tukey’s adjusted p = 0.0004).

As shown in Fig. 3, consistent with previous reports in the offspring of DIO rats, AgRP-IR was significantly decreased in the PVH from mice fed HFD for 8 weeks, a timepoint at which these animals are obese and leptin-resistant^19^,^22^, indicating that long-term consumption of an HFD profoundly disrupts this critical circuit (8 weeks HFD: 37.48 ± 13.8, n = 3, Tukey’s adjusted p = 0.0009). Since we found that even brief exposure to HFD was sufficient to alter AgRP/NPY neuronal output (Figs 1 and 2), we next determined whether short-term feeding of HFD also affected the density of AgRP-IR in the PVH. As shown in Fig. 3, after only 48 h of HFD feeding, AgRP-IR in the PVH was significantly reduced to the same degree as observed in both *ob/ob* and 8 weeks HFD mice (48 h HFD: 22.41 ± 7.4, n = 3/group, Tukey’s adjusted p = 0.0003), indicating that AgRP immunoreactivity in the PVH is diminished very early in the response to feeding a HFD.

The loss of AgRP-IR in the PVH may reflect a physical loss of axonal projections from ARH AgRP neurons to the PVH or, alternatively, an effect of HFD on the AgRP protein itself (e.g., defective axonal trafficking or post-translational processing of the peptide). To distinguish between these possibilities, we generated an AgRP-Cre-tdTomato mouse by crossing the AgRP-Cre transgenic mouse^37^, which expresses Cre recombinase under the control of the AgRP promoter with a tdTomato reporter mouse, in which the fluorescent protein tdTomato is expressed behind the strong *CAG* promoter at the ubiquitous Rosa26 locus^38^. Following Cre-dependent excision of a transcriptional blocker, tdTomato is strongly expressed only in AgRP neurons in these offspring. Because soluble tdTomato is expressed from a different locus by a different promoter, it is unlikely that tdTomato trafficking to the PVH is affected by a possible HFD-induced defect in AgRP trafficking or processing. Consistent with this, we observed a qualitatively similar decrease in red fluorescence in the PVH of AgRP-tdTomato mice following either 3 days or 3 weeks of HFD feeding (Fig. 3C), suggesting that the decreased AgRP-IR in the PVH is due to a loss of axonal projections.

The PVH is the principle target of AgRP neurons in the hypothalamus^39^,^40^, thus a significant loss of projections from ARH AgRP neurons may be reflected as a decreased synapse number in the PVH. To test this hypothesis, we performed immunohistochemistry for the synaptic marker synaptophysin (Syp), which is expressed by virtually all neurons in the brain and is widely used as an indirect measure of synaptic density^41^,^42^. As shown in Fig. 3D, along with the decrease in AgRP-IR and tdTomato fluorescence, there was also a significant decrease in the intensity of Syp-IR, suggesting that after only 3 days of high-fat feeding, there is a physical loss of synapses to the PVH.

### Diet composition, not calorie intake, induces persistent activation of AgRP neurons in HFD-fed mice

**Figure 4:**
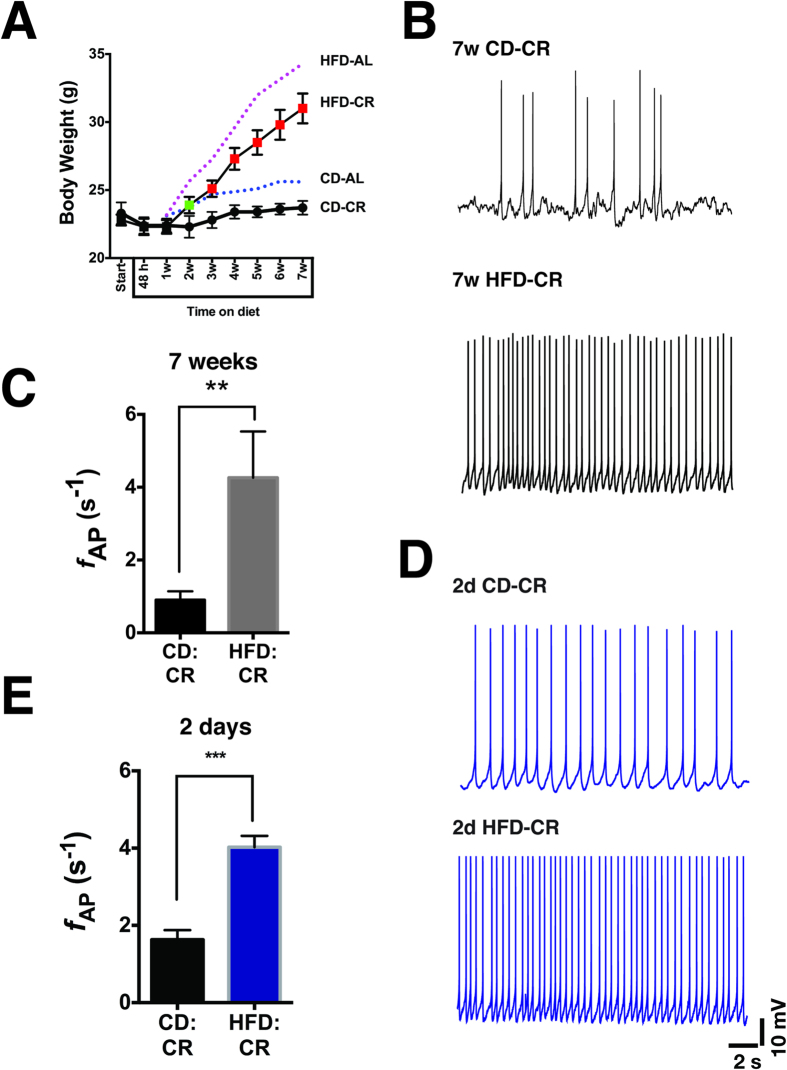
Diet composition alone can remodel the intrinsic electrical activity of ARH AgRP neurons. (**A**) Body weight curve for mice fed a calorie-restricted CD (CD-CR) or HFD (HFD-CR). Calorie restriction was begun at 6 weeks of age and continued for 7 weeks. The mean body weight of the HFD-CR mice was statistically greater than that of the CD-CR group by 2 weeks of HFD feeding (green symbol: p < 0.05; red symbols: p < 0.001). Body weight curves from *ad libitum* fed CD (CD-AL; blue dashed line) and HFD (HFD-AL; purple dashed line) are given for reference (data taken from Baver *et al.*, 2014). (**B**) Representative traces of spontaneous action potentials in an AgRP/NPY neuron from a 7 weeks CD-CR mouse and a 7 weeks HFD-CR mouse. Scale bars: 10 mV and 2 s. (**C**) Mean action potential firing rates from AgRP/NPY neurons from 7 weeks CD-CR and HFD-CR mice. **p = 0.0006, Mann-Whitney U test. (**D**) Representative traces of spontaneous action potentials in AgRP/NPY neurons from a 2 days CD-CR mouse and a 2 days HFD-CR mouse. Scale bars: 10 mV and 2 s. (**E**) Mean action potential firing rats from AgRP/NPY neurons from mice fed CD-CR (black) or HFD-CR (blue) for only 2 days. ***p = < 0.0001, Mann-Whitney U test.

In the early phase of high-fat feeding, mice exhibit hyperphagia^31^, thus in the first 1–2 weeks of HFD, mice consume both more dietary fat *and* more calories. It is therefore possible that the HFD-induced plasticity we observed in AgRP/NPY neurons is due at least in part to increased calorie consumption. To dissect the effect of increased dietary fat from increased caloric intake, we yoked a cohort of age-matched HFD-fed NPY-GFP mice (HFD-CR) to a group on CD diet (CD-CR) such that the HFD group was given the same daily calories as the CD group (~14 kcal/mouse/day). The CD group was also given ~14 kcal daily portion of food to control for the change from *ad libitum* to restricted feeding. As shown in Fig. 4A, even on a restricted calorie diet, mice fed HFD still gained a significant amount of body weight compared to the CD-CR group (note that the food-restricted CD group exhibits less age-related weight gain than *ad libitum* CD-fed mice), suggesting that diet composition alone is sufficient to alter energy balance in mice and promote weight gain. We next investigated whether consumption of an isocaloric, but high-fat diet altered electrical excitability in ARH AgRP/NPY neurons. As shown in Fig. 4B–D, just as in *ad libitum* HFD-fed mice, caloric restriction of an HFD induces a similar persistent activation of AgRP/NPY neurons (CD-CR: 0.8 ± 0.2, n = 8; HFD-CR: 4.3 ± 1.3, n = 7; p = 0.0006, Mann-Whitney U Test).

Since the HFD-CR mice still gained weight in spite of long-term calorie restriction, there remains the possibility that increased body weight and/or adiposity in these mice may contribute to the persistent increase in AgRP neuronal firing from these mice. Therefore, to control for the effects of diet, calories, and body weight, we fed a separate group of age-matched male mice HFR-CR for only 2 days, a time point at which HFD fed mice have not yet gained weight (Fig. 4A). CD mice were also calorie restricted for 2 days. As shown in Fig. 4A,B, even after only 2 days of restricted-calorie HFD, AgRP neuronal firing was significantly increased from 1.6 ± 0.25 s^−1^ (n = 24) in 2d CD-CR mice to 4.0 ± 0.3 s^−1^ (n = 28) in 2d HFD-CR mice (p < 0.0001), suggesting that diet composition alone can alter neuronal excitability and AP firing in ARH AgRP/NPY neurons.

## Discussion

In lean animals, AgRP/NPY neuronal firing is exquisitely sensitive to nutritional status and peripheral metabolic and nutritional signals: neuronal output increases in response to food deprivation^6^,^7^,^15^,^22^,^23^,^34^ and circadian timing^25^ and is potently inhibited by hormonal signals such as leptin^15^,^16^,^22^. We recently reported that long-term (>8 weeks) consumption of an HFD was associated with disruption of this intrinsic plasticity: AgRP/NPY neurons from DIO animals were persistently activated, regardless of nutritional status and refractory to inhibition by leptin^22^. In this study, our goal was to determine the time course of altered AgRP neuronal firing and leptin resistance following HFD exposure and determine the relative contribution of diet composition versus caloric intake to the onset of these defects in neuronal function. We found that the diet-dependent increase in AgRP neuronal output occurs very quickly, within 48 h of consuming HFD and that the composition of the diet is itself a significant contributor to the development of hypothalamic neuronal dysfunction, as we observed increased AgRP neuronal output in neurons from mice fed a defined calorie, high-fat diet.

Our finding that AgRP neurons from mice fed HFD for a short time were still robustly inhibited by leptin, despite their persistent diet-dependent activation was somewhat surprising, as several other studies have demonstrated that leptin-resistance occurs shortly after exposure to HFD, on a similar time scale to what we used here (2 days in ref. 30 and 6 days in ref. 27). However, those studies used biochemical assays of LepRb function to assess leptin sensitivity, namely upregulation of SOCS3 along with impairment of STAT3 phosphorylation, whereas we directly measured the leptin-dependent inhibition of neuronal firing in AgRP neurons. In our previous study, we found that the LepRb-mediated inhibition of AgRP neurons occurs at least in part due to modulation of K^+^ channels via a Src-family kinase^22^. Thus, it is possible that the signal transduction pathway that mediates leptin-dependent AgRP neuronal inhibition uses an alternative pathway from the JAK/STAT/SOCS3 pathway, raising the intriguing possibility that these effects are differentially sensitive to the impact of DIO-dependent increases in serum leptin. Our results also support the hypothesis that hypothalamic leptin resistance alone cannot account for the persistently increased firing of ARH AgRP neurons and that the increased excitability of these neurons must be due to something else, such as another hormone such as insulin or perhaps some aspect of the high-fat diet itself. One possible mechanistic explanation for our finding is that high-fat feeding, even on the short time scale described here, may cause disruption of mitochondrial dynamics, thus contributing to decreased neuronal excitability, as demonstrated by Dietrich, *et al.* (2013)^43^.

Similar to what we show here (Fig. 4A), Petro *et al.* demonstrated that mice pair-fed a high-fat diet still gained significant amounts of weight compared to control mice fed a low-fat diet^44^. Thus, dietary fat itself can cause obesity independently of caloric intake. Our results extend this finding to show that even caloric restriction of a HFD remodels the neurons that regulate appetite and that this remodeling occurs very quickly, within 48 h. Further, since the additional weight in the HFD-CR mice cannot be coming from increased intake, we postulate that it is due to a decrease in energy expenditure, highlighting the importance of AgRP neurons and the circuits they are a part of (e.g., the melanocortin system) in regulating not only food intake, but also energy expenditure.

In addition to the increased electrical activity of AgRP neurons in the ARH, we also found that anatomical projections of AgRP neurons from ARH to the PVH are reduced in both short- and long-term HFD-fed mice, suggesting these projections are altered in response even to acute changes in diet in the absence of body measureable weight changes. The neurotrophic action of leptin has been implicated in the development of ARH_AgRP_ → PVH projections, as these projections are significantly decreased in both *ob/ob* mice and offspring of DIO rats^35^,^36^. The mice used in our study were wild-type mice not genetically prone to obesity, demonstrating that the loss of ARH_AgRP_ → PVH projections can occur even in adult animals in response to an obesogenic challenge, highlighting the plasticity of these neurons and their sensitivity to energy balance signals.

In summary, we demonstrate here that changes in the intrinsic neuronal output and anatomical projections of ARH AgRP neurons occur rapidly following exposure to a high-fat diet and that these changes depend on the fat composition of the diet rather than caloric intake. Interestingly, although leptin resistance in the LepRb-JAK/STAT/SOCS3 pathway in AgRP neurons occurs within a similar timeframe as we have examined here, the inhibition of AgRP neuronal electrical activity is not yet leptin resistant, suggesting that the timing of diet modification may be an important consideration for therapeutic approaches to obesity.

## Methods

### Animal care

All animal care and experimental procedures were performed in accordance with a protocol approved by the Institutional Animal Care and Use Committee (IACUC) at the University of Tennessee Health Science Center (14-056.0). Mice were housed at 22–24 °C on a 12 h light/dark cycle (lights on at 6:00 AM). All electrophysiology studies described here used transgenic hrGFP-NPY mice in which humanized *Renilla* green fluorescent protein (hrGFP) is expressed under the control of the murine NPY promoter^45^. Experiments involving immunohistochemical analysis of ARH AgRP projections to the paraventricular nucleus of the hypothalamus (PVH) used age-matched non-transgenic littermates of the hrGFP-NPY mice, *ob/ob* mice, or C57Bl6/J mice. Adult (>8 weeks) male mice were used for all experiments. Mice fed HFD long-term (>6 weeks) were started on the high-fat diet at 6 weeks of age and maintained on HFD until they were 12–14 weeks old.

Control diet (CD) fed mice were fed a standard pelleted rodent chow (Teklad 7912, 17 kcal% fat, 3.1 kcal/g metabolizable energy). For some experiments, aged-matched littermates were fed a high-fat diet (HFD; D12451, 45 kcal% fat, 4.5 kcal/g metabolizable energy, Research Diets, Inc.), for 2–6 d. There was no significant difference in firing rate or leptin sensitivity in neurons from mice fed HFD up to 6 days, so all time points were collapsed into a single group and presented as “short-term HFD”, except for the immunohistochemistry experiments in which mice were fed HFD for either 48 h or 8 weeks. Both CD and HFD were administered *ad libitum* and water was freely available at all times. Mice that were fasted had all of the food removed from the cage just prior to the start of the dark cycle (6:00 PM) and were food-deprived for no more than 16 hours; water was freely available.

For experiments involving calorie restriction, we measured the daily food intake of CD-fed mice for one week (average daily intake/mouse ~14 kcal/day). A measured amount of food corresponding to ~14 kcal/day of either CD or HFD was added to each cage in the afternoon prior to “lights-off”, beginning at 6 weeks of age and continuing for 7 weeks. To control for the switch from *ad libitum* to restricted feeding, CD-fed mice were given ~14 kcal/day of CD. For all groups, water was freely available at all times. All mice were weighed weekly as well as just prior to use in experiments. In all experiments, mice were euthanized and slices prepared between 9 and 10 AM to minimize circadian variation in feeding.

### Electrophysiology

#### Slice preparation

Adult male mice (8–16 weeks old) were deeply anesthetized using isoflurane prior to decapitation and rapid removal of the brain. The brain was then immediately submerged in ice-cold, oxygenated cutting solution (in mM: 80 NaCl, 90 sucrose, 3.5 KCl, 4.5 MgSO_4_, 0.5 CaCl_2_, 1.25 NaH_2_PO_4_, 23 NaHCO_3_, and 10 glucose). The brain was blocked for sectioning and 250 μm coronal slices were cut using a Vibratome (VT1000S, Leica). Sections containing arcuate nucleus were incubated in oxygenated cutting solution for at least 1 h prior to recording.

#### Slice recording

Slices were transferred to a recording chamber constantly perfused (~2 ml/min) with oxygenated artificial cerebrospinal fluid (aCSF, in mM: 119 NaCl, 2.5 KCl, 1 MgSO_4_, 2.5 CaCl_2_, 1.25 NaH_2_PO_4_, 23 NaHCO_3_, and 10 glucose). Fast synaptic neurotransmission was blocked using 100 μM picrotoxin, 10 μM CNQX, and 20 μ D,L – AP5 to inhibit GABA_A_, AMPA-R and NMDA-R, respectively to isolate spontaneous, intrinsic action potentials in AgRP/NPY neurons. GFP-positive AgRP/NPY neurons were identified using epifluorescence and standard GFP filters on a fixed-stage Olympus BX-51WI microscope equipped with an XM-10IR CCD camera (Olympus America, Inc.). All recordings were performed using a Multiclamp 700B amplifier interfaced via a Digidata 1440 digitizer and controlled using Clampex 10 (Molecular Devices). Data were acquired at 5 Hz and digitized at 20 Hz using the built-in 4 pole Bessel filter of the Multiclamp. Pipette capacitance was nulled following the formation of a GΩ seal in all experiments.

Recording pipettes were prepared from filamented, thin-wall glass (TW150, World Precision Instruments) and had a resistance of 5–7 MΩ when filled with intracellular solution (in mM: 130 K-gluconate, 10 KCl, 0.3 CaCl_2_, 1 MgCl_2,_ 1 EGTA, 3 MgATP, 0.3 NaGTP, 10 Na-phosphocreatine, and 10 Hepes, pH = 7.35 with KOH). The liquid junction potential (LJP) between the aCSF and intracellular solution was measured to be 14.2 mV; membrane potential recordings were corrected for the LJP off-line. All current-clamp recordings were performed at 32–34 °C, and membrane potential and spontaneous action potentials were recorded for at least 10 minutes. Neurons that did not exhibit spontaneous activity within 2 minutes were not included in the analysis. Leptin (100 nM, National Hormone and Peptide Program) was bath applied for 60–80 seconds, after which perfusion with normal aCSF resumed. For experiments involving leptin, a stable baseline was acquired for 2–3 minutes prior to the addition of leptin.

### Immunohistochemistry

Three adult (>12 weeks old) male mice from each group (CD, *ob/ob*, 8 weeks HFD, 48 h HFD) were anesthetized with Avertin and transcardially perfused with 10% formalin. The ‘8 weeks HFD’ mice were started on HFD at 6 weeks of age and were 14 weeks old when sacrificed for IHC. Brains were removed and postfixed overnight at 4 °C in formalin. Coronal sections (50 μm) containing paraventricular nucleus of the hypothalamus (PVH) were cut on a vibratome (VT1000S, Leica). Sections were washed 3× for 15 min in PBS, then permeabilized and blocked in PBS +0.25% Triton X-100 +5% donkey serum +1% IgG- and fatty-acid free BSA (Jackson Immunoresearch). Slices were incubated in goat anti-AgRP antibody (1:1000, Santa Cruz Biotechnology) for 3 h at room temperature, then washed and incubated with donkey anti-goat Alexa568 secondary antibody (1:400, Invitrogen) for 2 h at room temperature, then mounted on glass slides for imaging.

Sections were imaged at 1024 × 1024 resolution using a Zeiss 710NLO confocal microscope. Optical section thickness, laser power, pixel dwell time, and detector settings were determined for the brightest section and then applied equally across all groups. All images were acquired using Zeiss ZEN software and offline image analysis was performed using the ZEN software and FIJI (ImageJ 2.0). An experimenter blind to the identity of the experimental groups performed image acquisition and analysis.

### Data analysis and Statistics

#### Electrophysiology

Action potential frequency was measured using Clampfit 10. Descriptive statistics and group differences were determined using Prism 6 (GraphPad). Action potential frequencies for Figs 1 and 2 were compared using a Kruskal-Wallis ANOVA with Dunn’s multiple comparisons *post hoc* test. Action potential frequencies for calorie-restricted groups in Fig. 4 were compared using a Mann-Whitney U Test. All data are presented as mean ± SEM. A value of p < 0.05 was considered significant.

#### Image analysis

To quantify AgRP immunoreactivity, three-dimensional confocal images containing PVH from approximately the same anatomical location were used per mouse for image analysis. Images were imported into FIJI and thresholded to generate a binary image (the same threshold value was applied to each image in the stack). The binary image was then skeletonized using the “Skeletonize” plugin included in FIJI to thin all objects above threshold to a line 1-pixel wide. Each image in the stack was skeletonized individually and the sum of the integrated fluorescence density of each skeletonized 2D image in the 3D stack used as the measure of AgRP immunoreactivity for each brain section. Group differences were determined using a one-way ANOVA with a Tukey’s multiple comparisons *post hoc* test. A value of p < 0.05 was considered significant. All image analysis was conducted blind to the experimental groups.

## Additional Information

**How to cite this article**: Wei, W. *et al.* Diet composition, not calorie intake, rapidly alters intrinsic excitability of hypothalamic AgRP/NPY neurons in mice. *Sci. Rep.* **5**, 16810; doi: 10.1038/srep16810 (2015).
